# Corrigendum

**DOI:** 10.1002/vms3.1107

**Published:** 2023-04-03

In Section 4.2.1 of the “Discussion,” the statement “The detection of A. capra in sheep in our study is the first report of A. capra DNA in sheep in the Mediterranean area and in Corsica” is wrong and should have read: “The report of *A. capra* in sheep in our studies confirm the result previously described in Corsica where this bacteria was found in sheep and goat with a seroprevalence of 30% and 13%, respectively (Jouglin et al., 2022).”

The reference: “Jouglin, M.; Rispe, C.; Grech‐Angelini, S.; Gallois, M.; Malandrin, L. *Anaplasma capra* in sheep and goats on Corsica Island, France: A European lineage within *A. capra* clade II? *Ticks and Tick‐Borne Disease*, 2022, *13*, 101934.” Should be add in the “References” section.

In addition, the conclusion section should read as below.

Our results confirm the extent of possible circulation of different pathogens near Corsican wetlands, not only in ticks collected from livestock but also directly in cattle and sheep, with two (Trypanosoma sp. and Babesia sp.) being detected for the first time in cattle and one for the first time in sheep.

We apologize for these errors.

**REFERENCES**

Defaye, B., Moutailler, S., Grech‐Angelini, S., Galon, C., Ferrandi, S., Pasqualini, V., & Quilichini, Y. (2022). Detecting zoonotic and non‐zoonotic pathogens in livestock and their ticks in Corsican wetlands. Veterinary Medicine and Science, 116. https://doi.org/10.1002/vms3.956

